# De Novo* PTEN* Mutation in a Young Boy with Cutaneous Vasculitis

**DOI:** 10.1155/2017/9682803

**Published:** 2017-04-24

**Authors:** Angela Mauro, Ebun Omoyinmi, Neil James Sebire, Angela Barnicoat, Paul Brogan

**Affiliations:** ^1^Department of Paediatrics, San Giacomo Hospital, Via Edilio Raggio, Novi Ligure, Italy; ^2^Infection, Inflammation, and Rheumatology Section, UCL Great Ormond Street Institute of Child Health, London, UK; ^3^Department of Histopathology, Great Ormond Street Hospital NHS Foundation Trust, London, UK; ^4^Department of Clinical Genetics, Great Ormond Street Hospital NHS Foundation Trust, London, UK

## Abstract

Phosphatase and tensin homolog (PTEN) is the protein encoded by the* PTEN* gene (10q23.3).* PTEN* mutations are related to a variety of rare diseases referred to collectively as PTEN hamartoma tumor syndromes (PHTS), which include Cowden Syndrome, Bannayan-Riley-Ruvalcaba syndrome, Proteus Syndrome, and Proteus-like syndrome. These diseases are associated with an increased risk of malignancy and for this reason an accurate and early diagnosis is essential in order to institute cancer surveillance. PTEN is a regulator of growth and homeostasis in immune system cells, although there are limited data describing immune dysregulation caused by* PTEN* mutations. We describe a case of PHTS syndrome caused by a de novo mutation in* PTEN* detected using a targeted next generation sequencing (NGS) gene panel which was instigated for workup of cutaneous vasculitis. We highlight the diagnostic utility of this approach and that mutations in* PTEN* may be associated with immune-dysregulatory features such as vasculitis in young children.

## 1. Introduction

Phosphatase and tensin homolog (PTEN) is the protein encoded by the PTEN gene, located on 10q23.3. PTEN is an important tumor suppressor and is one of the most commonly mutated tumor suppressor genes reported in sporadic human cancer [1]. PTEN dephosphorylates phosphatidylinositol(3,4,5)P3 (PIP3) to phosphatidylinositol 4,5-bisphosphate (PIP_2_), thus antagonizing pathways downstream of receptor tyrosine kinases and phosphatidylinositol-3-kinase (PI3K). PTEN acts as a tumor suppressor gene by negatively regulating PI3K-Akt and mammalian target of rapamycin (mTOR) signaling pathways, which play a critical role in cell proliferation and oncogenesis [2]. The class Ia PI3K family contains three catalytic isoforms (p110a, p110b, and p110d) encoded by three different genes, of which p110d is restricted to the immune system, while p110a and p110b are expressed in all tissues and organs [3, 4].

PTEN mutations are related to a variety of rare syndromes, collectively known as PTEN hamartoma tumor syndromes (PHTS), which include Cowden Syndrome (CS), Bannayan-Riley-Ruvalcaba syndrome (BRRS), and Proteus and related syndromes [5], although the latter association remains somewhat controversial [6, 7]. CS is inherited in an autosomal dominant fashion, although a proportion of cases arise as a result of new mutations. Its incidence is approximately 1 : 200,000, and the most frequently described mutations are point mutations, deletions, or insertions [8]. CS has a heterogeneous clinical presentation [9]; clinical features include multiple hamartomas, macrocephaly, typical mucocutaneous lesions (trichilemmomas), acral keratosis, glycogenic acanthosis of the oesophagus, and papillomatous papules. PTEN mutations can sometimes be associated with autistic spectrum disorders and developmental delay. It is also associated with thyroid, breast, renal, and endometrial manifestations, including cancer in all of these tissues. The lifetime risk of developing gastrointestinal carcinoma in CS is approximately 9% [10].

While most previous reports have focused on the tumor suppressor role of* PTEN*, it is increasingly recognized that immune dysregulation is also an important feature in some patients with* PTEN* mutations. We describe a sporadic case of possible PHTS associated with cutaneous vasculitis from early in life. This latter clinical manifestation prompted genetic screening with a targeted next generation sequencing (NGS) gene panel, called the “vasculitis and inflammation panel (VIP),” which screens for 166 monogenic immune-dysregulatory and autoinflammatory diseases. The patient's parents provided written consent for all of the investigations described in this report and the report itself.

## 2. Case Report

**Figure 1:**
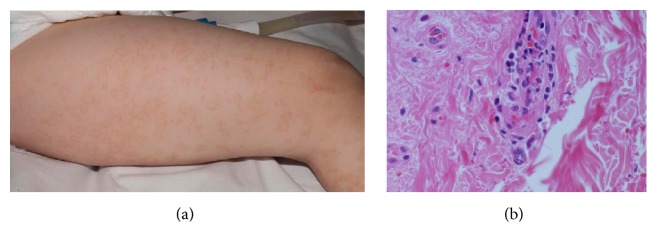
(a) Vasculitic rash with a distinct circinate pattern. (b) Photomicrograph of skin biopsy demonstrating inflammation affecting the superficial and deep dermal small vessels, comprising predominantly mononuclear inflammatory cells, but with focal destruction of the vessel walls with fibrin deposition, red cell fragmentation, and scattered nuclear fragments (haematoxylin and eosin, original magnification ×100).

A three-year-old Caucasian boy was investigated for diffuse vasculitic skin lesions (Figure 1(a)). His parents were unrelated; his mother was pregnant with the second child, and his family history was otherwise unremarkable. He was born by forceps delivery at 42 weeks following a pregnancy with normal antenatal scans. He walked independently at 19 months of age. His parents reported concerns that he had some social communication difficulties; a formal assessment (aged 4 years) confirmed the presence of autistic spectrum disorder. His past medical history included recurrent upper respiratory tract infections (URTI; approximately 8–10 per annum), including a severe episode of croup requiring brief admission to intensive care at the age of two years for airway support. Physical examination revealed macrocephaly (head circumference > 97.5th centile) and an unusual vasculitic rash with a distinct circinate pattern (Figure 1(a)). A skin biopsy revealed thrombotic, lymphocytic vasculitis with an absence of immune-complex deposition on immunofluorescence (Figure 1(b)). Blood tests revealed normal inflammatory markers (erythrocyte sedimentation rate, C reactive protein, and serum amyloid A); negative autoantibodies (ANA, ENA, and ANCA); normal renal and hepatic function; normal urinalysis; normal immunoglobulin levels; and mild lymphopenia (total lymphocytes 1.34 × 10^9^/L; reference range 2–9.5 × 10^9^/L). Routine fever gene Sanger sequencing for more commonly encountered autoinflammatory diseases known to cause skin rashes was negative for* MEFV*,* MVK*,* NLRP3*,* NOD2*, and* TNFRSF1A*. Tonsillectomy, undertaken for recurrent upper airway compromise secondary to intercurrent (presumed viral) infections, resulted in complete resolution of the cutaneous vasculitis, compatible with a reactive or “hypersensitivity” cutaneous lymphocytic vasculitis [11, 12]. The presence of macrocephaly, autistic spectrum disorder, recurrent URTI, and cutaneous vasculitis prompted us to screen him using a targeted next generation sequencing (NGS) panel, which targets known 166 genes broadly associated with immune dysregulation or vasculopathy, the vasculitis and inflammation panel [13].

## 3. Methods

The Agilent EArray online tool (https://earray.chem.agilent.com/suredesign/) was used to design an NGS panel targeting 166 genes, grouped into nine broad clinical phenotypes: autoinflammatory disease; monogenic vasculitis/vasculopathy; complement defects; monogenic lupus; haemophagocytic lymphohistiocytosis; early-onset inflammatory bowel disease; autoimmune lymphoproliferative syndromes; monogenic stroke; and hereditary amyloidosis. The targeted region includes coding exons, conserved noncoding exons, upstream promoter regions, and splice sites. Captured and indexed libraries (QXT Target Enrichment System) were sequenced as a multiplex of 16 samples on an Illumina MiSeq sequencer in paired-end mode. Read alignment, variant calling, and annotation were performed using Agilent Sure Call v3.0 software.

## 4. Results

VIP targeted sequencing revealed a mutation in the* PTEN* gene, NM_000314; c.650T>A, p.V217D, which encodes for phosphatase and tensin homolog protein, dominant mutation of which is associated with CS [14], and related entities. This was confirmed with Sanger sequencing and predicted to be damaging by 3 widely used computational tools: SIFT, PolyPhen2, and MutationTaster [15–17]. No other class 4 or class 5 genetic variants were detected. Neither parent had this variant (screened with Sanger sequencing), consistent with a de novo mutation in the index case. His (now born) male sibling had normal development at age 6 months and normal head circumference and has thus not been screened for this mutation.

## 5. Discussion

We report a case of possible PHTS caused by heterozygous mutation in* PTEN* characterized by the presence of social communication difficulty, macrocephaly, recurrent URTI, and early-onset cutaneous vasculitis, using a targeted NGS panel (VIP).

Chen et al. recently described the immune dysregulation in patients with PHTS. Seventy-nine patients with pathogenic germline* PTEN* mutations underwent detailed immunological studies, which demonstrated the presence of T- and B-cell dysfunctions such as dysregulation, lymphopenia, increased transitional B-cell counts, and reduced CD4/CD8 ratio [18].

*PTEN* is an important tumor suppressor gene but also plays a role in immune maturation and function. The p.V217D* PTEN *mutation we identified has previously been described as causing CS [14] and accounts for the macrocephaly and autistic spectrum disorder; although recurrent severe URTI have been described anecdotally in PHTS [19], whether or not the* PTEN* variant directly accounts for recurrent infection in our patient remains speculative. Similarly, we also cannot conclude definitively that the reactive lymphocytic vasculitis we observed was caused by immune dysregulation associated with this* PTEN* mutation, although it is increasingly recognized that PTEN plays an important role in the immune system (and it was the reason we included this gene in our VIP gene panel). Indeed both immunodeficiency and autoimmunity are lesser-known features in some patients with CS and related conditions [19, 20]. Furthermore, the vasculitis resolved completely following tonsillectomy, compatible with an aberrant host immunological response to intercurrent infection. Since immunodeficiency has been described in a few cases of CS, and* PTEN *is an important regulator of B-, T-, and NK-cell function, we speculate that lymphocytic vasculitis was probably linked to this genetic mutation, although the exact mechanism remains uncertain. Thus this hypothesis is plausible but at the moment unconfirmed in our patient.

Browning et al. described two cases of CS with recurrent URTI and B- and T-cell dysregulation [19]. The first case was a five-year-old boy with macrocephaly and mild developmental delay associated with heterozygous mutation (c.203A>G, p.Y68C) of* PTEN*. Immunological investigations showed pan-hypogammaglobulinemia, including absence of antibodies to HIB despite vaccination, although he was able to mount a short-lived protective antibody response to unconjugated pneumococcal vaccine. Peripheral blood lymphocyte immune-phenotyping revealed increased numbers of T-, polyclonal-B-, NK-, CD5 and CD10 B-cells. Western blot analysis in peripheral blood T-cells demonstrated a low PTEN level and increased Akt and S6 phosphorylation following stimulation with anti-CD3 or anti-CD28. After activation, the patient's T-cells had a higher levels of PIP_3_ [19]. The second case was a ten-year-old boy with heterozygous nonsense mutation (c.87T>A) in* PTEN* which encodes a STOP codon in place of a tyrosine at amino acid position 29 in the PTEN protein, with macrocephaly and recurrent URTI. Lymphocyte immune-phenotype profile showed mild CD4 and CD8 lymphopenia but normal levels of B- and NK-cells [19].

Suzuki et al. studied how PTEN influences the regulation of B-lymphocytes in B-cell-specific* PTEN*-mutated mice [21]. Murine PTEN-deficient B cells were resistant to apoptotic and migratory stimuli and demonstrated defective immunoglobulin class switching, emphasizing a role for PTEN in the regulation of B cells [21]. Furthermore, mice with T-cell-specific deficiency in PTEN have dysregulated thymic T-cell proliferation and excessive T-cell activation, which contributed to not only the development of tumors, but also autoimmunity in this animal model [22]. PTEN is also a negative regulator of cytolytic function in NK-cells. A study of PTEN expression and its function in human and murine NK-cells demonstrated that immature NK-cells with low cytolytic activity have higher PTEN expression than mature NK-cells and that a decrease in PTEN activity causes an increase in cytolytic NK-cell function [23]. Since dominant gain-of-function mutations in* PIK3CD* cause humoral immunodeficiency, lymphopenia, recurrent URTI, and lymphoid hyperplasia [24, 25], and PTEN regulates PI3K [26], it has been suggested that heterozygous mutation of* PTEN* results in immune dysregulation which shares some features of PI3K*δ* immunodeficiency. This latter syndrome is characterized by increased catalytic function of PI3K*δ* that induces a primary immunodeficiency, with similar albeit more severe clinical presentation than the immunodeficiency described in CS [27].

There are limited reports of autoimmunity in CS; Heindl et al. described a series of 34 patients with* PTEN* mutations with miscellaneous autoimmune or immune-dysregulatory manifestations including gastrointestinal lymphoid hyperplasia, hyperplastic tonsils (reminiscent of our case), thymus hyperplasia, lymphocytic thyroiditis, autoimmune haemolytic anaemia, and colitis [20]. To the best of our knowledge, our case is the first report of possible PHTS with vasculitis, investigation of which ultimately led to the correct molecular diagnosis. Thus far, tonsillectomy has been a successful treatment for our patient, by reducing severity and frequency of URTI and resulting in complete resolution of the cutaneous vasculitis; however the diagnosis of PHTS has important lifetime consequences due to an increased lifetime cancer risk, irrespective of minor autoimmune sequelae. Thus lifelong surveillance for cancer is mandatory. Tan et al. suggest that paediatric patients with* PTEN *mutations under 18 years of age should undergo annual targeted history and physical examinations from diagnosis, including baseline thyroid examinations with ultrasound, and additional bespoke cancer screening programmes for males and females into adulthood [10].

In conclusion, this case report emphasizes the diagnostic utility of NGS, which resulted in the diagnosis of PHTS caused by sporadic heterozygous mutation in* PTEN*, providing a unifying molecular cause to explain seemingly unrelated and diverse clinical features. The immunological features of CS and other PTEN related diseases (immunodeficiency +/− autoimmunity) may be the initial presenting feature in young children, as illustrated by our patient. While the cutaneous vasculitis we observed has a seemingly benign prognosis (thus far), PHTS carries an increased lifetime risk of malignancy, and thus timely diagnosis is essential to institute lifelong cancer surveillance [28]. We acknowledge that this case does not prove a causal relationship of this* PTEN* variant and reactive vasculitis but does emphasize an emerging hypothesis regarding a possible link between PTEN and immune dysregulation worthy of future study.
